# Vascular Disrupting Agent Arsenic Trioxide Enhances Thermoradiotherapy of Solid Tumors

**DOI:** 10.1155/2012/934918

**Published:** 2012-01-04

**Authors:** Robert J. Griffin, Brent W. Williams, Nathan A. Koonce, John C. Bischof, Chang W. Song, Rajalakshmi Asur, Meenakshi Upreti

**Affiliations:** ^1^Department of Radiation Oncology, University of Arkansas for Medical Sciences, 4301 W. Markham Street, Slot No. 824, Little Rock, AR 72205, USA; ^2^Department of Mechanical Engineering and Therapeutic Radiology, University of Minnesota, 240 Delaware Street, SE Slot No. 494, Minneapolis, MN 55455, USA

## Abstract

Our previous studies demonstrated arsenic trioxide- (ATO-) induced selective tumor vascular disruption and augmentation of thermal or radiotherapy effect against solid tumors. These results suggested that a trimodality approach of radiation, ATO, and local hyperthermia may have potent therapeutic efficacy against solid tumors. Here, we report the antitumor effect of hypofractionated radiation followed by ATO administration and local 42.5 °C hyperthermia and the effects of cisplatin and thermoradiotherapy. We found that the therapeutic efficacy of ATO-based thermoradiotherapy was equal or greater than that of cisplatin-based thermoradiotherapy, and marked evidence of *in vivo* apoptosis and tumor necrosis were observed in ATO-treated tumors. We conclude that ATO-based thermoradiotherapy is a powerful means to control tumor growth by using vascular disruption to augment the effects of thermal and radiation therapy.

## 1. Introduction

As_2_O_3_ (ATO) has now been widely studied and used successfully as a therapeutic agent for acute promyelocytic leukemia, as it causes differentiation and apoptosis of leukemic cells [1–6]. This FDA-approved compound has also demonstrated significant potential as a solid tumor antivascular and angiogenesis inhibiting agent in preclinical studies. We were among the first groups to discover the potent and selective tumor vascular disruption caused by this agent [7]. Subsequently we have demonstrated significant thermosensitization of tumors and an increase in tumor response to radiation therapy induced by ATO treatment [8–10]. Although there have been numerous reports in the literature, relatively little is clear about the cellular and physiological mechanisms of ATO effect on solid tumors *in vivo*, especially in regard to antivascular effects of the drug and how to use these effects to design effective clinical regimens [7, 11–14].

From our earlier findings, we concluded that selective tumor vascular shutdown caused by ATO induced boldly demarcated necrotic regions. We also observed an increase in mean or median tumor oxygenation values for up to a week after ATO therapy [9, 10, 15]. These studies, and the low toxicity caused by ATO in our experience, suggested that a regimen consisting of radiation, ATO, and local hyperthermia may be more effective than the already clinically used cisplatin-based combination therapy.

Phase III clinical studies using thermoradiotherapy with cisplatin have begun [16]. This clinical trial was expanded to a multicenter, international trial against cervical cancer due to the encouraging initial results. More than 15 different Phase I, II, or III studies to compare the effects of cisplatin/radiation and heat/cisplatin/radiation have been performed in the past 20 years [17]. However, use of ATO, a vascular disrupting agent with a different mechanism of action than conventional cisplatin therapy and which may be effective in patients resistant or insensitive to platinum-based regimens, could be a better candidate for the treatment of solid tumors. The possible advantages of a tumor and stromal- (i.e., vascular-) targeted multimodality approach to improved cancer treatment are discussed in the context of using ATO or similar vascular disrupting agents to sensitize the tumor to thermoradiotherapy.

## 2. Materials and Methods

### 2.1. Cell Line

**FSaII Tumor**

This fibrosarcoma of C3H mice (Jackson Laboratories) was originally obtained from Dr. Herman Suit (Massachusetts General Hospital). Stock cells are stored in liquid nitrogen, and new cultures are established every 2-3 months. FSaII tumor cells grow well in RPMI-1640 medium supplemented with 10% bovine calf serum.

### 2.2. Tumor Induction

FSaII tumor cells in exponential growth phase were harvested using 0.25% trypsin in Hepes-buffered medium, washed, and counted. A subcutaneous injection of 2 × 10^5^ cells in 0.05 mL serum-free medium was made in the hind thigh of female C3H mice.

### 2.3. Arsenic Trioxide

An i.p. injection of 4–8 mg/kg arsenic trioxide (ATO or Trisenox^TM^, Cephalon Oncology, Inc., Frazer, Pa, USA) was performed by using a clinical grade 1 mg/mL stock solution for each mouse, and imaging or treatment was performed at specific times after this injection. Control mice were injected with an equal volume of phosphate-buffered saline, pH 7.4.

### 2.4. Cisplatin

Doses of 2 or 6 mg/kg cisplatin (Sigma-Aldrich, St. Louis, Mo, USA) were administered i.p. in saline at the frequency indicated for each of the studies comparing to ATO treatment.

### 2.5. Window Chamber Tumor Growth and Intravital Microscopy

Skin-fold chambers made of anodized aluminum frames were surgically implanted into a fold of dorsal skin in female nu/nu mice. Briefly, the dorsal skin was sandwiched between two identical anodized round aluminum frames. The 19 mm × 22 mm chamber was held fixed on the mouse by three screws between the frames. The skin was also attached to the chamber with 4-O silk. The skin on both sides of the viewing region was removed, exposing the dermis containing the microvasculature. Excess fascia on the dermis was removed to assist clear visualization of the microvasculature. Windows milled from quartz glass microslides (Chase Scientific Glass, Rockwood, Tenn, USA) were used to cover the vascular area. The distance between the windows was maintained at 450 *μ*m by a spacing gasket on the inside of the frames, leaving room for seeded tumor to grow. 1-2 × 10^6^ tumor cells were added in 30 uL of matrigel just before placement of the glass windows. Treatments and imaging were performed over the course of tumor growth and treatment as described [18].

### 2.6. FLIVO Reagent

FLIVO (FAM-VAD-FMK, 50 *μ*g per vial, Immunochemistry Technologies, LLC, Bloomington, Minn, USA) was dissolved in 50 *μ*L of DMSO and diluted by the addition of 200 *μ*L of sterile PBS, pH 7.4. At 30 min after an i.v. injection of 0.1 mL of FLIVO cell permeant probe via the lateral tail vein, fluorescent images were captured at 20X using a Hamamatsu C2400 camera (Hamamatsu, Japan) and Broadway Imaging Software (Data Translation, Marlboro, Mass, USA) on an Eclipse TE200 bench-top microscope (Nikon, Japan).

### 2.7. Histological Analysis

Tumor-bearing mice were sacrificed, and the tumor was removed at the specified time point after treatment. The tissue was fixed in 10% neutral buffered formalin, and, after processing and embedding, tissue sections at 5 *μ*M were prepared and stained with hematoxylin and eosin. An Olympus BX40 microscope was used to image multiple fields at 20x magnification which were then fit together using Adobe Photoshop.

### 2.8. X-Irradiation

Tumors were locally irradiated with 5 Gy per fraction by a Philips 250 Kv X-ray machine at a dose rate of 1.4 Gy/min. The body was shielded with lead with only the tumor and foot exposed to the X-ray beam.

### 2.9. Hyperthermia

Tumors were heated by immersing the tumor-bearing legs of anesthetized mice into preheated water for 60 min as described previously [8]. The water temperature and the temperature of tissues were routinely measured with needle-type (29 gauge) thermocouples. The thermoprofiles in tumors and normal tissues during water-bath heating have been thoroughly studied in our group [8]. The temperature in the tumors during water bath heating is consistently 0.3–0.5°C below the water temperature. All temperatures quoted refer to the water temperature during hyperthermia treatment.

### 2.10. Assessment of Tumor Growth

Tumor volume was measured with a caliper (Scienceware) and calculated according to the equation: (*a* ^2^ × *b*)/2, where “*a*” is the width and “*b*” the length of the tumor.

## 3. Results

**Figure 1:**
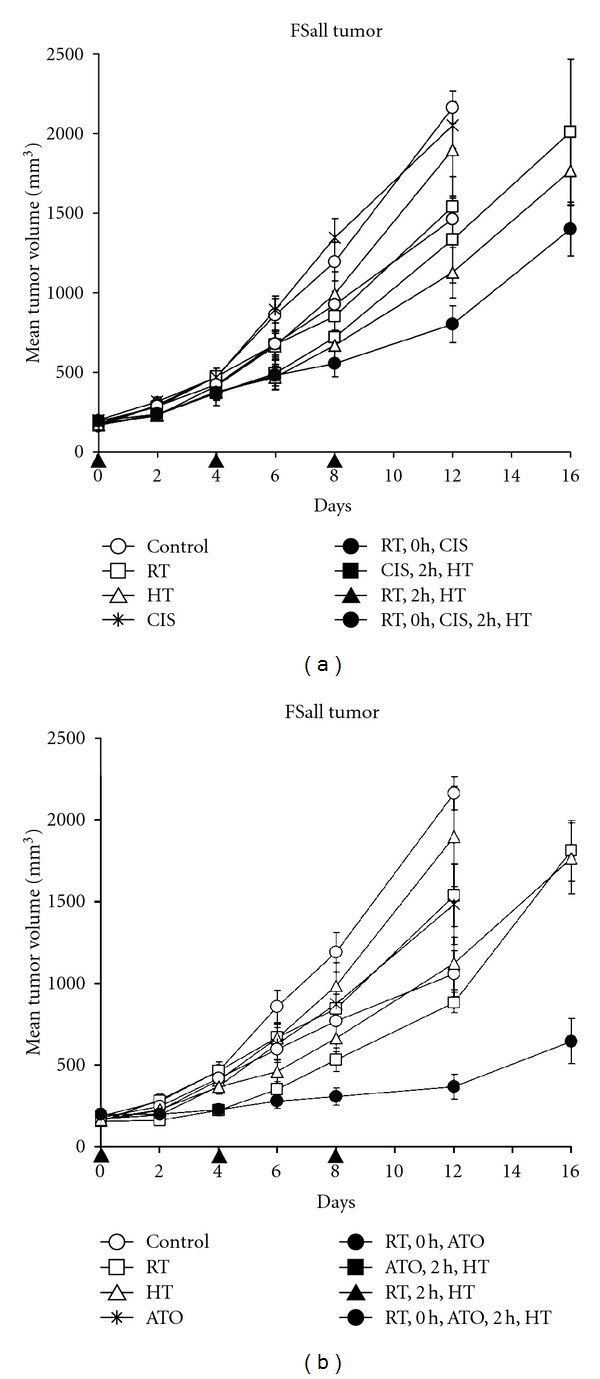
Comparison of arsenic trioxide and cisplatin enhanced thermoradiotherapy of FSaII fibrosarcoma tumors applied every 4 days as indicated by the arrows on the *x*-axis. Cisplatin was given at 2 mg/kg, while ATO was given at 8 mg/kg. Radiation fractions were 5 Gy, and hyperthermia was applied at 42.5°C (60 min). In the dual- or trimodality treatment groups, radiation was given first, followed by ATO or cisplatin injection within 30 min and hyperthermia was applied beginning 2 h after the completion of radiation. *N* = 5–7 mice per group. Errors bars represent 1 SEM.

As shown in Figure 1(a) cisplatin at 2 mg/kg i.p. and Figure 1(b) ATO at 8 mg/kg i.p. given every 4 days for a total of three treatments were able to significantly improve the antitumor effect of radiation combined with hyperthermia against FSaII tumors. The single modalities were only slightly effective, and the dual-modality combinations further improved the tumor growth delay, but the trimodality treatments were clearly the most effective in delaying tumor growth, especially in the case of ATO where an approximate 2-fold increase in growth delay compared to any other treatment occurred (*P* < 0.001 by day 8 of therapy onward). Cisplatin-based thermoradiotherapy was only significantly different from the dual-modality treatments by day 16 after start of treatment (*P* = 0.03).

**Figure 2:**
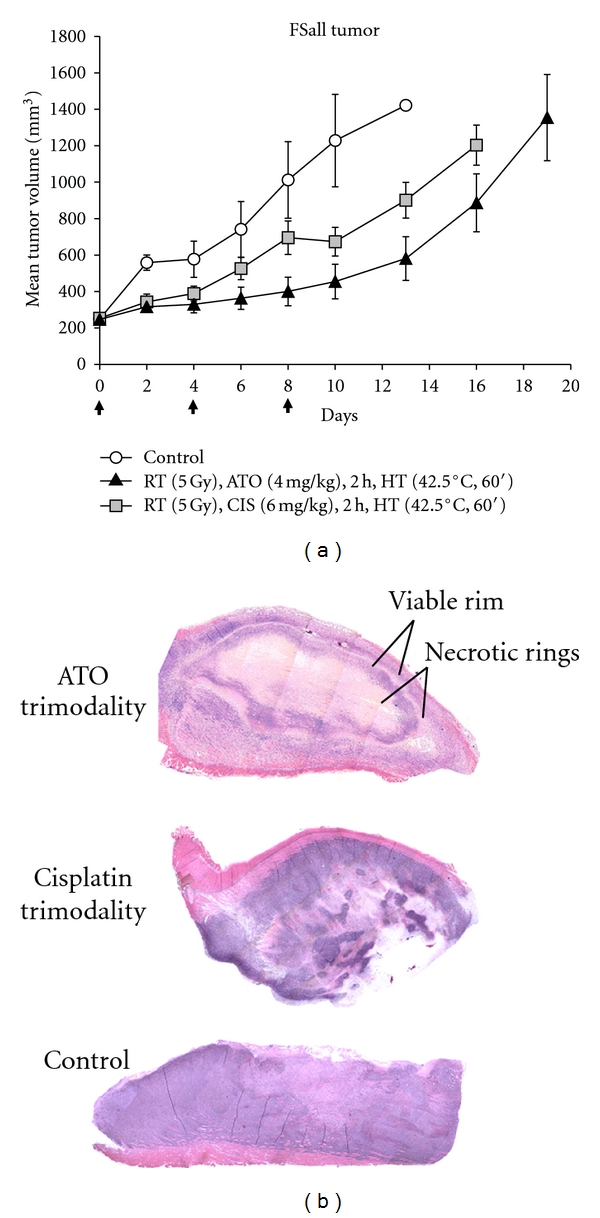
Equimolar comparison of arsenic trioxide and cisplatin as part of a recurring trimodality regimen every 4 days against FSaII fibrosarcoma tumors. As in the previous figure, in the dual- or trimodality treatment groups, radiation was given first, followed by ATO or cisplatin injection within 30 min and hyperthermia was applied beginning 2 h after the completion of radiation. *N* = 6–8 mice per group. Error bars represent 1 SEM. INSET: histological evidence of antitumor effects of repeated trimodality therapy applied every 4 days, 3 times in total in FSaII tumors (tumors taken from treatment groups on the last day of measurement) compared to control tumor.

In order to more definitively understand the potential of ATO as a part of a trimodality regimen, we did a subsequent study using an equimolar amount of cisplatin or ATO (4 mg/kg ATO MW: 198 versus 6 mg/kg cisplatin MW: 300, resp.). In this study, ATO was again found to be more effective than cisplatin in augmenting the effects of radiation and hyperthermia on tumor inhibition (*P* = 0.02 versus cisplatin-based therapy by day 10 after start of treatment). As shown in Figure 2, ATO maximally increased tumor growth delay by a factor of 2.5-fold as compared to control tumor growth, while cisplatin increased the tumor growth delay response by a factor of about 2-fold on average compared to control tumors in this group of animals.

A typical composite image of the entire tumor gross section from an untreated tumor and from tumors treated with either cisplatin-based or ATO-based trimodality therapy is shown in Figure 2 (inset). The effects of repeated radiation, arsenic trioxide, and heat treatments are clearly evident in the marked rings of live and dead regions of tissue in the ATO-treated mouse, while the cisplatin-based therapy indicates there were more widespread regions of viable tumor remaining after treatment and the control tumor shows homogenous staining of live cells with very little architectural variation. The images shown are the typical results obtained from groups of 3 control or trimodality-treated tumors that were prepared for histology and studied.

**Figure 3:**
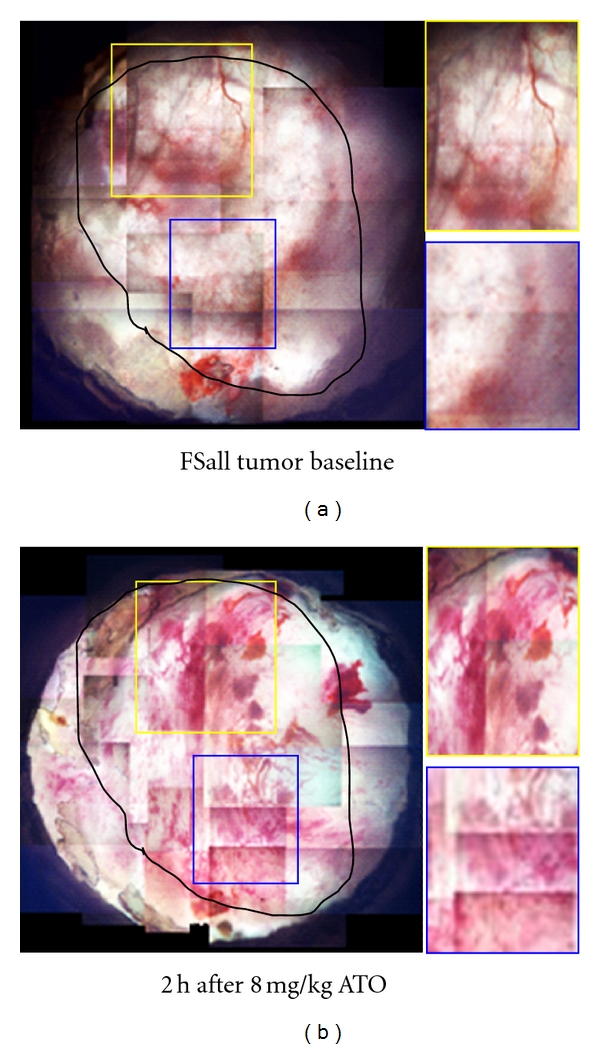
Representative window chamber imaging of 8 mg/kg i.p. arsenic trioxide-induced vascular damage in FSaII fibrosarcoma grown in nude mice. FSaII tumor was implanted and 7 days later the tumor was imaged before and 2 h after an i.p. injection of 8 mg/kg ATO. The tumor is outlined in black and the boxes have been placed in the areas where typical marked evidence of vascular damage occurred.

**Figure 4:**
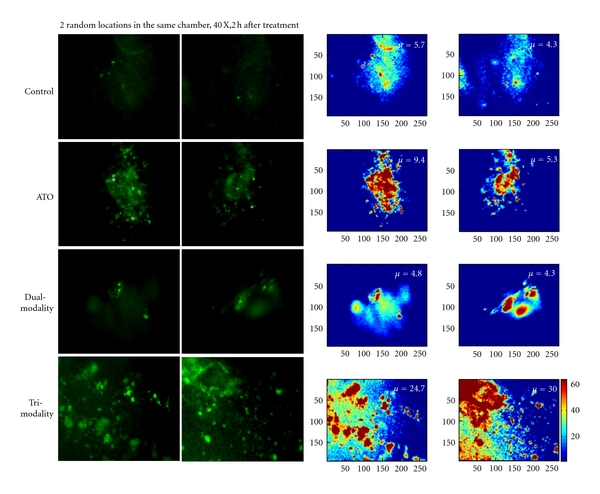
ATO, heat, and radiation induce apoptotic activity by 2 h after combined treatments in tumor of treated mouse. *In vivo* imaging of polycaspase inhibitor in FSaII tumor grown in the window chamber and treated with 8 mg/kg arsenic trioxide, thermoradiotherapy (dual-modality, 5 Gy followed by 42.5°C (30 min) 2 h later), or arsenic trioxide combined with thermoradiotherapy (trimodality; 5 Gy, 8 mg/kg ATO, 2 h, 42.5°C (30 min). The right insets are intensity plots of the images on the left, created in Matlab. *μ*: mean pixel intensity for all pixels in each image. The color bar represents the relative intensities of individual pixels.

We subsequently investigated the *in vivo* cellular and physiological mechanisms by which ATO selectively damages solid tumor and increases the effect of hyperthermia and radiation. We employed the dorsal skin-fold window chamber (DSFC) technique for intravital imaging of tumor tissue [18–21] to visualize real-time changes in tumor physiology and vascularity. As shown in Figure 3, ATO causes substantial and noticeable vascular damage in relatively large areas of the tumor by 2 h after i.p. injection of tumor bearing mice. A fluorescent pan-caspase probe (FLICA or FLIVO Poly- (pan) caspase detection) was used for *in vivo* assessment of cell death and viability, as we previously reported [21].  Figure 4 shows changes in the amount of apoptosis occurring in FSaII tumors grown in the window chamber in control tumors, 2 h after a single dose of 8 mg/kg ATO i.p., 2 h after 5 Gy followed by 30 min of 42.5°C heating or 2 h after 5 Gy and 8 mg/kg ATO i.p. followed by 42.5°C heating, as detected by the polycaspase inhibitor. There appeared to be increased apoptotic activity in the representative regions of the tumor in the mouse treated with ATO alone as well as the mouse that received trimodality treatment compared to control tumor or tumor treated with heat and radiation. An intensity plot was made for each image (Figure 4), and the overall mean pixel intensity was calculated for each group. Upon statistical analysis of the mean intensity values, the trimodality mean pixel intensity was found to be significantly increased compared to control, ATO alone, or heat combined with radiation exposure (*P* < 0.03). ATO treatment alone appeared to visually cause more apoptosis than that in tumor treated with heat combined with radiation or in control tumor, but this was not found to be statistically significant.

## 4. Discussion

The results of the current study suggest that fractionated radiation in concert with arsenic trioxide and clinically achievable thermal doses is a viable option against tumors that may not be adequately treated by mono- or dual-therapy regimens or may be insensitive to conventional cisplatin-based chemotherapy. Our results agree with a number of other intriguing studies employing a variety of trimodality approaches, some with other antivascular or antiangiogenic agents [22–30]. In view of these results and the clinical benefit obtained with trimodality approaches involving more traditional therapeutics [16, 17, 26, 31, 32], continued development and implementation of this treatment strategy appears to be promising. 

Many previously studied triple-combination regimens do not include hyperthermia, but instead pair an antiangiogenic or other biological targeting agent with more standard chemotherapy and radiation [25, 33]. These type of studies are intriguing, but it should be noted that they are quite different from studies employing thermal therapy since the mechanism of action and rationale for sequencing will likely be very different for each strategy. Work with the vascular-targeting agent combretastatin has been highly encouraging and supports the intelligent use of agents that target the tumor vasculature in heat-based multimodality regimens [29, 30, 34, 35]. However, the authors of these preclinical and clinical studies all highlight the critical need for well-controlled clinical trials where the sequencing of the agents is of utmost importance. These studies and our rationally designed study based on known synergism of ATO, radiation, and hyperthermia strongly suggest that careful attention to how treatments are combined will maximize the benefits for patients.

Our earlier work had demonstrated enhancement of radiation-induced growth delay when ATO was given before or after radiation. However, since we knew that tumor blood flow was rapidly reduced or abolished in certain regions of the tumor upon ATO administration, it appeared logical to avoid induction of hypoxia before radiation by administering ATO after each radiation fraction. The application of heat to the tumor was elected to be after the radiation and the ATO injection for two reasons. First, although there may be a benefit in increasing the tumor oxygenation and thus the tumor radiosensitivity when heat is applied prior to radiation, we had observed a more significant thermosensitization when heat was applied after ATO injection at the point of greatest blood flow shutdown. Secondly, it is well known that radiation damage repair is inhibited by heating after radiation exposure. Therefore, the combination of ATO-induced tumor and endothelial thermosensitivity and potential inhibition of DNA damage repair were expected to cause the greatest improvement in radiation-induced tumor growth delay. Indeed, the tumor growth was suspended in some animals during the period of therapy, suggesting that continued therapy may even obtain tumor cures using this multimodality approach. It is possible that other sequences and/or frequencies of treatment may be even more potent in these models. The important result is that arsenic trioxide, as a novel vascular disrupting agent, can be effectively added to a thermoradiotherapy regimen that may have certain advantages and synergy compared to regimens employing traditional chemotherapy.

We further studied the effects of ATO alone or in combination with heat and radiation therapy on tumor blood flow and apoptosis *in vivo* by employing intravital microscopy and histological analysis. A current focus of our group is to delineate the kinetics of vascular cell apoptosis and tumor cell apoptosis after these treatments. Ultimately, apoptosis detection *in vivo* could be used as a surrogate marker of treatment response in clinical situations. Overall, we have clearly demonstrated here, and previously, that ATO can be a powerful radiation- or thermosensitizing agent [8, 9]. Others have also recently found significant effects of ATO therapy on the tumor control obtained by thermal ablation or fractionated radiation therapy [11, 36]. Newer agents based on trivalent arsenicals that may selectively target tumor endothelium have reached clinical trials, and; thus, there is reason to believe that acceptable clinical doses of arsenic-based therapy would be achievable in the clinic and/or better delivery strategies will be available and contribute to positive therapeutic outcomes [13, 37, 38].

Equal or better antitumor activity of arsenic trioxide-based thermoradiotherapy was observed in our study compared with a cisplatin-based regimen. The study with cisplatin at equimolar ratio to ATO (Figure 2) was particularly enlightening in that it allows us to hypothesize that ATO has the potential to improve upon currently employed trimodality therapy against cervical cancer. Out of the several antivascular strategies currently being tested either preclinically or in clinical trials, it seems likely that at least one of these agents will become a realistic option to the clinician wanting to maximize the effects of a thermoradiotherapy treatment plan.

## 5. Conclusions

Clinical trial development of an arsenical-based trimodality strategy may be warranted, especially in view of the wealth of clinical experience already accumulated with arsenic against leukemia, lymphoma, and other solid tumors. The continued success of several radiation-, chemotherapy- and hyperthermia-based trials suggest that there is much to be gained with agents that selectively target and disrupt solid tumor vasculature and angiogenic capability with different, yet complementary, mechanisms of action such as ATO, thermal therapy, and radiation therapy.
